# Cigarette smokers' intention to quit smoking in Dire Dawa town Ethiopia: an assessment using the Transtheoretical Model

**DOI:** 10.1186/1471-2458-10-320

**Published:** 2010-06-08

**Authors:** Eshetu Girma, Tsion Assefa, Amare Deribew

**Affiliations:** 1 Department of Health Education and Behavioral Sciences, Jimma University, Jimma, Ethiopia; 2 Department of Epidemiology, Jimma University, Jimma, Ethiopia

## Abstract

**Background:**

Cessation of smoking reduces morbidity and mortality related to tobacco smoking. It is essential to explore the intention of individuals to quit smoking to design effective interventions. The objective of this study was to assess cigarette smokers' intention to quit smoking in Dire Dawa town using the Transtheoretical model.

**Methods:**

From February 15 to 19, 2009, we conducted a community based cross-sectional study among 384 current cigarette smokers in Dire Dawa town east Ethiopia. Data was collected by trained personnel using a pretested structured questionnaire. The data was analyzed using SPSS version 16.0.

**Results:**

Two hundred and nineteen (57%) smokers in the study area had the intention to quit cigarette smoking within the next six months and all the process of change had an increasing trend across the stages. Based on the Fragestrom test of nicotine dependence of cigarette, 35 (9.1%), 69 (18%) and 48(12.5%) were very high, high and medium dependent on nicotine respectively. For the majority 247(64.3%) of the respondents, the mean score of cons of smoking outweighs the pros score (negative decisional balance). Only 66(17.2%) had high self efficacy not to smoke in places and situations that can aggravate smoking.

**Conclusions:**

Majority of the smokers had the intention to quit smoking. All the process of change had an increasing trend across the stages. Those who had no intention to quit smoking had high level of dependence on nicotine and low self efficacy. The pros of smoking were decreasing while the cons were increasing across the stages. Stage based interventions should be done to move the smokers from their current stage to an advanced stages of quitting cigarette smoking.

## Background

There are more than 4,000 different compounds in tobacco smoke. More than 40 of the chemicals in tobacco are known to cause cancer [1]. Nicotine is the drug in tobacco that makes smoking a powerful addiction. Experts rank nicotine ahead of alcohol, cocaine and heroin with regard to the severity of dependence resulting from its use. Tobacco dependence is also recognized as a disease in the World Health Organization's International Classification of Diseases (ICD-10) and the American Psychiatric Association's Diagnostic and Statistical Manual (DSM-IV) [2,3]. In developed countries a large proportion of smokers want to stop smoking and many try to stop, but the corresponding proportions in developing countries are low. Quit rates are also low in many developing countries. Smokers who try to quit often find it difficult because of the addictive properties of nicotine [4]. Based on Ethiopian Demographic and Health Survey (EDHS) 2005 report, among men with the age range of 15-49 years in Ethiopia, 9% of them smoke cigarettes and 5% consume other forms of tobacco. Even though, there is no complete data on the prevalence of smoking among women, EDHS shows that less than 2% of women in Ethiopia smoke cigarette [5]. While the urgency of preventing onset of smoking is generally understood, it is less well understood that cessation of smoking among those who have started to smoke is more important to reduce smoking related deaths. The most immediate health gains, therefore, must be achieved by persuading and helping existing smokers to quit [6-10]. However, there are few studies concerning cigarette smoking in Ethiopia and the intention of current smokers to quit smoking is not known in the country.

According to the Transtheoretical Model (TTM), without any planned intervention, an individual will remain stuck in the early stages of behavioral change. If an individual does not have a plan to change his/her behavior, there will be no motivation to change. However, if a person is motivated to change his/her behavior, there are specific processes and principles of change that can be applied during certain stages of change if progress through the stages is to occur [11].

**Figure 1:**
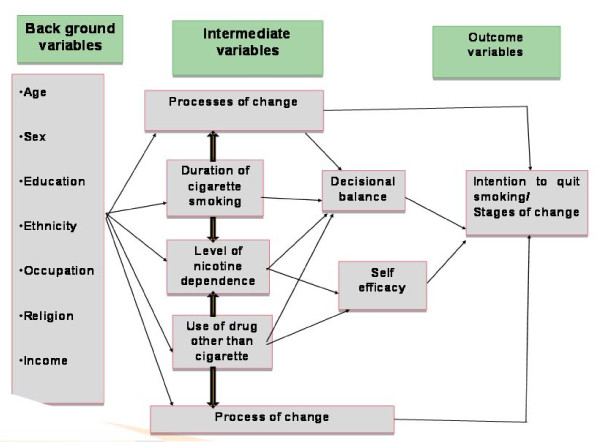
**Conceptual framework showing factors associated with intention to quit smoking**.

Considerable empirical evidences also support the transtheoretical model as a promising model to predict smoking cessation readiness. The main constructs of the model includes: the sequential stages of change; processes which people typically use to facilitate change; decisional balance, which predicts whether change will occur; and self efficacy, the persons' confidence they can make changes [11,12]. In this particular study a conceptual frame work was adopted by adding variables like the level of nicotine dependence, use of drugs other than cigarette, duration of cigarette smoking and the socio-demographic variables based on different research findings (figure 1).

## Methods

From February 15 to 19, 2009, a community based cross-sectional study was conducted to assess the intention of cigarette smokers to quit cigarette smoking in Dire Dawa town. Dire Dawa is located in the eastern part of Ethiopia 515 km from the capital city of Addis Ababa. The inhabitants are mainly merchants followed by government and non-government works. It has a total population of 342,827 [13]. The study population consisted of 384 adults who have ever smoked at least 100 cigarettes and were still smoking daily or occasionally for the last one month before the survey was conducted. The study participants were recruited by convenient sampling method from proportionally allocated and randomly selected cafeterias, hotels, bars, street, Khat (natural stimulant from Catha edulis plant)houses, shops and other work places in Dire Dawa town till the required sample size was obtained.

Data was collected by trained personnel using an Amharic (local language) version structured questionnaire adopted from Carlo Diclemente. Stages of change were measured by using the staging algorithm developed by Prochaska, Diclemente, and colleagues. The precontamplation, contemplation and preparation, stages were relevant to this study. Precontemplators were smokers who were not seriously thinking about quitting smoking in the next 6 months. If the respondents reported that they were thinking about quitting in the next 6 months but were *not *planning to quit in the next 30 days or were planning to quit in the next 30 days but had not yet made a 24-hour quit attempt within the past year, they were categorized in the contemplation stage. People who were thinking about quitting smoking over the next 30 days and had made a quit attempt in the last year were categorized in preparation stage. Process of change, self efficacy and decisional balance [14] constructs were also used.

Process of change of smoking is measured by five dimensions: consciousness raising, dramatic relief, environmental re-evaluation, social liberation, and self re-evaluation. Twenty Likert scale questions (1 = never, 2 = seldom, 3 = occasionally, 4 = often, 5 = repeatedly) were used to assess the five dimensions. The mean score of the five dimensions (process of changes) was re-coded as low (<3), medium (3) and high (>3).

Smoking self-efficacy is the person's level of confidence that he or she can manage temptations for not smoking in different places and situations that can reinforce smoking. Twenty Likert scale questions (1 = Not at all confident, 2 = Not very confident, 3 = moderately confident, 4 = Very confident, 5 = extremely confident) were used to assess challenging situations of individuals not to smoke cigarette. Individuals were categorized as having low, intermediate and high self-efficacy if they had mean score of <3, 3 and >3 respectively.

Smoking decisional balance is the balance between the perceived advantages of cigarette smoking behavior (the pros), and the perceived disadvantages of cigarette smoking behavior (the cons). Twenty Likert scale items (10 items for pros and 10 for cons of smoking) were administered to rate the level of importance of each item to make a decision about whether to smoke or not. The total score was re-coded as positive, undecided and negative based on the result of total pros minus total cons score of smoking. Six items questionnaire of Fagerstrom test for nicotine dependence was used to assess the level of nicotine dependence. It was measured by summing six factors: time to first cigarette, ability to refrain from smoking in forbidden areas, cigarettes smoked per day, smoking when ill, early morning smoking, and which cigarette would be most difficult to give up. Scores range from 0 to 10. The test of nicotine dependence scores were classified as: very low dependence (score 0-2), low dependence (score 3-4), medium dependence(score 5), high dependence(score 6-7), and very high dependence (score 8-10[15]. The internal reliability (coefficient alpha) of the Fagerstrom test of nicotine dependence in this study was 0.67.

Data was checked for completeness and analyzed using SPSS 16.0 statistical software. Descriptive analysis was done to see the distribution of socio-demographic characteristics, process of change and rate of intention to quit cigarette smoking. Pearson's chi-square test was done to see the association between process of change, and level of nicotine dependence with intention to quite cigarette smoking. To identify the predictors of intention to quite cigarette smoking, multivariate logistic regression analysis was done. Ethical approval was obtained from the Ethical Committee of Jimma University. Informed consent was obtained from the participants.

## Results

### Characteristics of the study participants

Among the 384 study population, 355 (92.4%) were male and the rest 7.6% were female. The majority of the respondents 180(46.9%) were in the age ranges of 20-29. The mean and median age of the respondents was 30.95 (SD = 10.55) and 28 years respectively (Table 1).

**Table 1:** Distribution of the socio-demographic characteristics of current cigarette smokers in Dire Dawa town, Ethiopia, February; 2009.

Socio-demographic characteristics	n (N = 384)	(%)
**Sex**		
Male	355	92.44
Female	29	7.56
**Religion**		
Muslim	206	53.64
Orthodox	151	39.32
Others (protestant, catholic Jhova, and no religion)	27	7.03
**Marital status**		
Single	211	54.94
Married	108	28.13
Divorced	44	11.46
Widowed	21	5.47
**Age (years)**		
15-19	30	7.8
20-29	180	46.9
30-39	106	27.6
40 and above	68	17.7
Educational status*		
illiterate	23	6.0
Lierate	361	94.0

*Educational status (illiterate = who could not read and write any language at all, literate = who can read and write any language)

### Cigarette Smoking Behaviors and Related History

The mean age of initiation of cigarette smoking was 22.15 (SD ± 6.99) years. The average length of smoking was 8.79(SD ± 8.34) years. Despite the relapse, 230 (59.9%) ever made a 24 hour quit attempt of cigarette smoking. The daily consumption of cigarettes ranged from 10 to 31. As the number of cigarette smoked and number of quit attempt increases, the probability of quitting smoking would increase (p-value < 0.001).

### Stages and Process of Change

Of the total participants, 165 (43.0%) did not have the intention to quit cigarette smoking at least within the next six months. Educated individuals were more likely to quit smoking than the illiterates (p = 0.029). In all the five process of change, individuals in the precontemplation stages had lower mean score of processes of change (mean of Consciousness raising = 9.15, dramatic relief = 8.54, environmental reevaluation = 10.55, Social liberation = 8.34, self reevaluation = 9.07) than individuals in the contemplation and preparation stages. The overall total mean scores i.e. all the process of change had an increasing trend across the stages.

### Level of Nicotine Dependence

of the total respondents, 27.1% had high and very high nicotine addiction level. Among the addicted individuals, 37.5% wanted to quit cigarette smoking. From the fragestrom test of nicotine dependence items, 34.6% of the total respondents answered yes for the question "do you smoke cigarette even if you are so ill that you are in bed most of the day?"

### Decisional Balance to Quit Smoking

For the majority 247(64.3%) of the respondents, the mean score of cons of smoking outweighs the pros score (negative decisional balance) whereas for 119(31%) of them, the mean score of pros of smoking outweighs the cons (had positive decisional balance).

### Self Efficacy not to Smoke Cigarette

The confidence of the smokers not to smoke on different places and situations that can aggravate smoking behavior demonstrated that only 66(17.2%) had high self efficacy where as 130(33.9%) had low confidence not to smoke. The majority 188 (49%) were in an intermediate situation not to smoke on those places and circumstances.

Individuals who had high consciousness rising score were 2 times more likely to have the intention to quit cigarette smoking than individuals with low consciousness, [OR = 2.0,(95%CI: 1.0,3.9)]. Participants in the high dramatic relief process of change were 3 times more likely to quite cigarette smoking than their counterparts, [OR = 3.2,(95%CI: 1.6, 6.5)]. There was no statistically significant difference among low, medium and high environmental and social liberation process of change with intention to quit cigarette smoking. Individuals with high self reevaluation were 2.6 times more likely to have the intention to quit smoking than low self reevaluation individuals, [OR = 2.6, (95%CI: 1.5, 4.5)]. As the degree of decisional balance and level of nicotine dependence increases, the likelihood of quitting cigarette smoking decreases. The remaining dependent and independent variables did not have significant statistical association with the intention to quit cigarette smoking (Table-2).

**Table 2:** Relationship of the covariates of stages of change/inention to quit cigarette smoking of current cigarette smokers in Dire Dawa town, Dire Dawa, Ethiopia, February; 2009

Variable	Intension to quit*		AOR (95.0% C.I)
		
	No	Yes	
		
Consciousness raising (CR)			
Low CR	137 (48.9)	143 (51.1)	1
Medium CR	7 (23.3)	23 (76.7)	5.04 (1.73, 14.71)
High CR	21 (28.4)	53 (71.6)	2.03 (1.04, 3.95)
Dramatic relief (DR)			
			
Low DR	138 (48.4)	147 (51.6)	1
Medium DR	9 (40.9)	13 (59.1)	1.49 (0.52, 4.28)
High DR	18 (23.4)	59 (76.6)	3.22 (1.59, 6.52)
Environmental reevalution (ER)			
			
Low ER	98 (51.3)	93 (48.7)	1
Medium ER	19 (55.9)	15 (44.1)	0.46 (0.19, 1.14)
High ER	48 (30.2)	111 (69.8)	1.07 (0.62, 1.87)
Social liberation (SL)			
			
Low SL	83 (48.8)	87 (51.2)	1
Medium SL	26 (40.6)	38 (59.4)	1.22 (0.60, 2.48)
High SL	56 (37.3)	94 (62.7)	0.66 (0.37, 1.19)
Self reevaluation (SR)			
Low SR	120 (55.0)	98 (45.0)	1
Medium SR	12 (37.5)	20 (62.5)	1.26 (0.54, 2.97)
High SR	33 (24.6)	101 (75.4)	2.59 (1.47, 4.55)
Self efficacy (SE)			
Low SE	73 (56.2)	57 (43.8)	1
Intermediate SE	72 (38.3)	116 (61.7)	1.78 (1.02, 3.09)
High SE	20 (30.3)	46 (69.7)	1.95 (0.91, 4.16)
Decisional balance (DB)			
Negative DB	76 (30.8)	171 (69.2)	1
undicided	6 (33.3)	12 (66.7)	1.05 (0.34, 3.30)
Positive DB	83 (69.7)	36 (30.3)	0.21 (0.12, 0.37)
Level of nicotine dependece			
V. low dependence	37 (27.8)	96 (72.2)	1
Low dependence	41 (41.4)	58 (58.6)	0.58 (0.31, 1.10)
Medium dependence	22 (45.8)	26 (54.2)	0.51 (0.22, 1.14)
High dependence	42 (60.9)	27 (39.1)	0.26 (0.12, 0.54)
V. high dependence	23 (65.7)	12 (34.3)	0.23 (0.09, 0.57)

*Intention to quit (yes = contemplation and preparation stages, No = precontamplation phase)

## Discussion

This study demonstrated that only 230 (59.9%) individuals have ever tried to quit smoking. Among those who ever tried to quit, 74.8% were in the contemplation and preparation stages of change. The majority of those who were relapsed cases of smoking had future intention to quit. Epidemiological data suggest that more than 70% of the 50 million smokers in the United States by 2000 have made at least one prior quit attempt, and approximately 46% try to quit each year [16]. Studies showed that the vast majority of individuals who have relapsed, approximately 85% of all smokers, will cycle back to the contemplation or preparation stage [17]. Such discouraging statistics have led many health professionals in the USA to report that they feel ineffective in the interventions of tobacco use which is also considered as a failure to appreciate the chronic nature of cigarette smoking behavior [16].

Majority of the study subjects generally had the intention to quit cigarette smoking within the next six months. This result is relatively higher than the finding observed in Australia (45.6%)[18] but lower than the report of the Irish survey (76%) [19]. A study in Swiss, among Current smokers revealed that 54% were precontemplators, 36% were contemplators and 4% were in the preparation stage [20].

Individuals who do not have the intention to quit cigarette smoking were the least active in using the processes of change, and those who intended to quit were the most active. The processes of change of patterns supported the notion that individuals who were in preparation stage were actively modifying their smoking habit. Individuals who were in the contemplation stage were gathering information and evaluating their smoking habit, and those in the precontamplation stage were doing the least across all the processes change.

Smoking is addictive, and addictions can override logical thinking processes, giving rise to thoughts that justify continued smoking and minimize reasons for quitting. Nicotine addiction leads to the unfortunate situation where an otherwise rational, motivated, knowledgeable person who understands the risks of tobacco, continues to use it. Studies have repeatdly shown that the more dependent a person is on nicotine, the more difficulty they have in quitting [21]. Based on the Fagestrom test of nicotine dependence on cigarette, 35(9.1%) were very high dependent, 69(18%) high and 48(12.5%) medium dependent on nicotine. This is high compared to a study in Turkey. The level of nicotine addiction by using Fagerstrom test were high and very high dependent on nicotine (18.2%) [22].

This study is the first of its kind in Ethiopia to assess the intention to quit smoking and the associated factors. However, the study has some limitations. First, due to the cross sectional nature of the study, individuals who were in the action and maintenance stages of change were not included. Second, convenient sampling makes the study participants non-representative of the general population.

## Conclusion

Majority of the smokers had the intention to quit smoking. All the process of change had an increasing trend across the stages. Those who had no intention to quit smoking had high level of nicotine dependence and low self efficacy. The pros of smoking were decreasing while the cons were increasing across the stages. Thus, stage based interventions should be taken to raise awareness, clarify misconceptions, develop unfavorable feelings, acknowledge the cons of smoking and to increase confidence on oneself to quit smoking through quit skill training and setting norms not to smoke in public places.

## Competing interests

The authors declare that they have no competing interests.

## Authors' contributions

EGK designed the study, analyzed the data and drafted the manuscript. TAB was involved in the design and reviewed the article. AD was involved in the design, analysis of the data and critically reviewed the article.

All authors read and approved the final manuscript.

## Pre-publication history

The pre-publication history for this paper can be accessed here:

http://www.biomedcentral.com/1471-2458/10/320/prepub
